# Mitochondrial Dysfunction Pathway Networks and Mitochondrial Dynamics in the Pathogenesis of Pituitary Adenomas

**DOI:** 10.3389/fendo.2019.00690

**Published:** 2019-10-09

**Authors:** Na Li, Xianquan Zhan

**Affiliations:** ^1^Key Laboratory of Cancer Proteomics of Chinese Ministry of Health, Xiangya Hospital, Central South University, Changsha, China; ^2^Hunan Engineering Laboratory for Structural Biology and Drug Design, Xiangya Hospital, Central South University, Changsha, China; ^3^State Local Joint Engineering Laboratory for Anticancer Drugs, Xiangya Hospital, Central South University, Changsha, China; ^4^National Clinical Research Center for Geriatric Disorders, Xiangya Hospital, Central South University, Changsha, China; ^5^Department of Oncology, Xiangya Hospital, Central South University, Changsha, China

**Keywords:** mitochondrial dysfunction, mitochondrial dynamics, pituitary adenomas, omics, systems biology

## Abstract

Mitochondrion is a multi-functional organelle, which is associated with various signaling pathway networks, including energy metabolism, oxidative stress, cell apoptosis, cell cycles, autophagy, and immunity process. Mitochondrial proteins have been discovered to modulate these signaling pathway networks, and multiple biological behaviors to adapt to various internal environments or signaling events of human pathogenesis. Accordingly, mitochondrial dysfunction that alters the bioenergetic and biosynthetic state might contribute to multiple diseases, including cell transformation and tumor. Multiomics studies have revealed that mitochondrial dysfunction, oxidative stress, and cell cycle dysregulation signaling pathways operate in human pituitary adenomas, which suggest mitochondria play critical roles in pituitary adenomas. Some drugs targeting mitochondria are found as a therapeutic strategy for pituitary adenomas, including melatonin, melatonin inhibitors, temozolomide, pyrimethamine, 18 beta-glycyrrhetinic acid, gossypol acetate, Yougui pill, T-2 toxin, grifolic acid, cyclosporine A, dopamine agonists, and paeoniflorin. This article reviews the latest experimental evidence and potential biological roles of mitochondrial dysfunction and mitochondrial dynamics in pituitary adenoma progression, potential molecular mechanisms between mitochondria and pituitary adenoma progression, and current status and perspectives of mitochondria-based biomarkers and targeted drugs for effective management of pituitary adenomas.

## Introduction

Pituitary adenomas are intracranial tumors that develop in the pituitary gland, and account for 10 to 25% of all intracranial neoplasms. Most pituitary adenomas are benign, nearly 35% sufferers present invasiveness and just 0.1–0.2% are diagnosed as carcinomas (1). Pituitary adenomas are commonly divided into functional pituitary adenomas, and non-functional pituitary adenomas according to the clinical level of hormone secretion (2). Functional pituitary adenomas are hormone-secreting pituitary adenomas that could cause hyperpituitarism, such as Cushing's syndrome, acromegaly, and hyperprolactinaemia; and non-functional pituitary adenomas are non-hormone-secreting pituitary adenomas (3). Pituitary adenomas are also divided into microadenomas (<10 mm) and macroadenomas (≥10 mm) according to tumor size (4). The clinically chief complaints of pituitary adenomas are visual field defects, headache, and increased intracranial pressure, which are usually derived from a compression of the neighboring tissues and structures. Another type of clinical problem is an inappropriate hormone secretion in hormone-secreting pituitary adenomas (5).

Currently, high-throughput omic technologies have been extensively used to study pituitary adenomas (6) from a multi-parameter systematic biology angle to overcome the irrationality that use a single molecule as biomarker for accurate predictive, preventive, and personalized medicine (PPPM) practice, because numerous molecules alter at the different levels of DNAs (genome), RNAs (transcriptome), proteins (proteome), and metabolites (metabolome), and are involved in different pathway network systems for tumorigenesis (7). Among the field of multiomics, transcriptomics, and proteomics are two important ways to systematically study the functions of genes (8, 9). Thousands of differentially expressed genes have been identified in human pituitary adenomas (10–12), which have addressed at a certain degree the functions of genes. However, transcriptiomics study cannot fully reveal the functions of genes, because proteins are the final performer of the corresponding genes, there are lots of regulations and modifications occurred in the process from mRNA to proteins. The number of proteins is much more than the number of transcripts, and the correlation coefficient only reaches 0.4 between proteomics and transcriptomics (13, 14). Therefore, proteomics is a more important way to address the functions of genes, especially subcellular proteomics such as mitochondrial proteomics is an effective method to reveal the specialized functions of a organelle to associate with a given diseases such as cancer (15). Currently mitochondrial proteomics has become a research hotpot because mitochondria are ubiquitously subcellular organelles responsible for providing energy to eukaryotic cells, and are the key links of metabolism, oxidative stress, cell apoptosis, cell cycles, autophagy, and immunity process (16), which are involved in a wide range of diseases including cancers (17). This review article will mainly focus on the mitochondrial dysfunction pathway alterations in pituitary adenomas from a systematic viewpoint.

**Figure 1:**
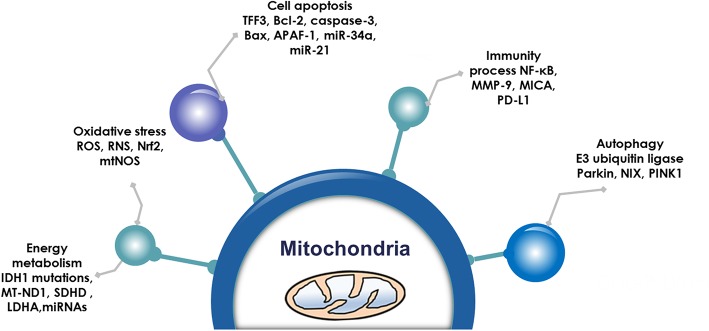
Mitochondrial functions. Emerging data show that mitochondria are associated with energy metabolism, oxidative stress, cell apoptosis autophagy, and immunity process in pituitary adenomas.

Pituitary adenoma proteomics-based molecular network study have revealed that mitochondrial dysfunction, oxidative stress, cell cycle dysregulation, and MAPK signaling abnormality are significantly associated with the pathogenesis of pituitary adenomas (18–21). Mitochondria are actually center of oxidative stress, which clearly demonstrate that mitochondrial dysfunction pathway plays important roles in pituitary adenomas. Furthermore, electron microscopy morphology study demonstrates that mitochondria are abundantly filled in cytoplasm of pituitary oncocytoma cells (22–24). Some studies demonstrate that the volume of mitochondria is different among different subtypes in pituitary adenomas; for example, the volume of mitochondria of prolactinoma is larger than acromegaly (25). More important are that some drugs targeting mitochondria have been reported as a therapeutic strategy for pituitary adenomas (Table 1) (26–39), including melatonin, melatonin inhibits, temozolomide and pyrimethamine, 18 beta-glycyrrhetinic acid, gossypol acetate, Yougui pill, T-2 toxin, grifolic acid, and paeoniflorin. Those evidences clearly demonstrate the important roles of mitochondrial biological functions and dynamic shift in pituitary adenoma pathogenesis, however, their molecular mechanisms remain unclear yet (2, 40). Mitochondria-based study might provide new insights into molecular mechanisms of pituitary adenomas, discover new biomarkers and molecular targets for effective management of pituitary adenomas. This review article discusses observations in the context of how mitochondrial dysfunction can influence the biological status in pituitary adenoma, including energy metabolism, oxidative stress, cell apoptosis, autophagy, and immunity (Figure 1; Table 2).

**Table 1:** Some drugs targeting mitochondria as a therapeutic strategy for pituitary adenomas.

**References**	**Drug**	**Testing index**	**Mechanisms**	**Species**
Yang et al. (26)	Melatonin	Caspase-3 activity, Bax mRNA expression, cytochrome c protein expression, Bcl-2 mRNA expression, and mitochondrial membrane potential.	Inhibits cell growth and increases cell apoptosis	Rat
Wang et al. (27)	Melatonin inhibitor	The activities of mitochondrial respiratory complexes, and the production of ATP.	Induces apoptosis	Rat
Dai et al. (28)	Temozolomide and pyrimethamine	Cell cycle arrest, DNA damage, cytochrome c release from mitochondria into cytosol, the expression of cathepsin B and Bax, decreased expressions of Bcl-2, MMP-2 and MMP-9, cleaved PARP, and phosphorylated histone H2AX as well as caspase3/7, 8, and 9 activities.	Inhibit proliferation, invasion and induce apoptosis of pituitary adenoma cell lines	Rat/mouse
Wang et al. (29)	18beta-glycyrrhetinic acid	Cell damage, cell viability, lactate dehydrogenase release, reactive oxygen species (ROS) and Ca(2+) concentration, G0/G1 phase arrest, apoptosis rate, mitochondrial membrane potential, a ratio of B cell lymphoma 2 (Bcl-2) and Bax, calcium/calmodulin-dependent protein kinase II (CaMKII), c-Jun N-terminal kinase (JNK), and P38.	Inhibits proliferation, and induces apoptosis	Rat
Tang et al. (30)	Gossypol acetate	Expressions of Bcl-2 and miR-15a.	Inhibits cell growth	Rat
Ji and Geng (31)	Yougui pill	The number of apoptotic cells, mRNA expressions of cytochrome c, caspase-3, caspase-9, and Bcl-2.	Mitochondria-mediated apoptosis pathway	Rat
Zhou et al. (32)	T-2 toxin	Intracellular NO and antioxidant enzyme activity, DeltaPsim, morphometric changes of mitochondria, the caspase pathway, and inflammatory factors.	Induces cell apoptosis	Rat
Zhao et al. (33)	Grifolic acid	Cellular ATP levels and the intracellular NAD/NADH ratio.	Induces cell death	Rat
Zhang et al. (34)	T-2 toxin	Reactive oxygen species (ROS), mitochondrial membrane potential, percentage of apoptotic cells, expression of p53, the activation of caspase-3, G1 cell population, mRNA and protein expressions of p16 and p21, cyclin D1, CDK4.	Induces cell apoptosis	Rat
Wei et al. (35)	Paeoniflorin	Protein expressions of cleave caspase-9, caspase-3, Bax, and Bcl-2, and phosphorylated p53.	Inhibits cell proliferation, and induces cell apoptosis	Rat
Deyu et al. (36)	T-2 toxin	Reactive oxygen species (ROS), DNA damage, the mitochondrial membrane potential, the superoxide dismutase (SOD) activity, expressions of glutathione peroxidase 1 (GPx-1), catalase (CAT), mitochondria-specific SOD-2, mitochondrial uncoupling protein-1, -2, and -3 (UCP-1, 2, and 3), adenosine triphosphate (ATP) levels, mitochondrial complex I activity, and the expressions of most of mitochondrial electron transport chain subunits, the expressions of mitophagy-specific proteins NIP-like protein X (NIX), PTEN-induced putative kinase protein 1 (PINK1), and E3 ubiquitin ligase Parkin.	Causes cell apoptosis	Rat
Kim et al. (37)	Cyclosporine A (CsA)	CsA induced a dose-dependent increase in expression of the autophagy markers LC3-I and LC3-II. Cell viability decreased significantly with increasing CsA concentrations largely due to an increase in apoptosis, with the changing level of Bcl-2 and Bax.	Induction of apoptotic or autophagic cell death i	Rat
Leng et al. (38)	Dopamine agonists	Dopamine receptor D5 activation increased production of reactive oxygen species (ROS), inhibited the MTOR pathway, induced macroautophagy/autophagy, and led to autophagic cell death (ACD) *in vitro* and *in vivo*.	Induced macroautophagy/autophagy	Human pituitary tumor cell
Wang et al. (39)	Bromocriptine (BRC) and artesunate (ART)	Low-dose ART combined with BRC synergistically inhibited the growth of GH3 and MMQ cell lines, caused cell death, attenuated cell migration and invasion, and suppressed the expression of extracellular prolactin. The induction of apoptosis after co-treatment was confirmed by immunofluorescent staining, assessment of caspase-3 protein expression, and flow cytometry.	Induction of apoptosis	Rat

**Table 2:** Mitochondrial dysfunction pathway in the pathogenesis of pituitary adenomas.

**References**	**Biological process**	**The related molecules**	**Function mechanism**	**Species**
An et al. (41)	Energy metabolism	Lactate dehydrogenase A (LDHA)	LDHA suppresses glucose uptake, lactate secretion, invasion and proliferation.	GH3 cells
Casar-Borota et al. (42)	Energy metabolism	Isocitrate dehydrogenase (IDH) 1 and 2	Mutant IDH1 and IDH2.	Human tissue specimen
Hao et al. (43)	Energy metabolism	Isocitrate dehydrogenase 1 (IDH1)	Somatic IDH1 mutation.	Human tissue specimen
Porcelli et al. (44)	Energy metabolism	Hypoxia inducible factor 1 subunit alpha(HIF1A)	A high frequency of homoplasmic disruptive mutations implicates disassembly of respiratory complex I *in vivo* which in turn contributes to the inability of oncocytic tumors to stabilize HIF1alpha.	Human tissue specimen and cell
Xekouki and Stratakis (45)	Energy metabolism	Succinate dehydrogenase (SDHx)	Loss of heterozygosity at the SDHD locus.	Human tissue specimen
Xekouki et al. (46)	Energy metabolism	Succinate dehydrogenase (SDH)	SDHD mutation.	Human tissue specimen and rats
Wu et al. (47)	Energy metabolism	Hsa-mir-181a-5p	Prolactin signaling pathway, and mitochondria related calcium reabsorption.	Human tissue specimen
Feng et al. (48)	Energy metabolism	14-3-3η protein	14-3-3η is exclusively overexpressed in oncocytomas, and 14-3-3η is capable of inhibiting glycolysis, leading to mitochondrial biogenesis in the presence of rotenone. In particular, 14-3-3η inhibits LDHA by direct interaction in the setting of complex I dysfunction.	Human tissue specimen and cell
Wang et al. (29)	Oxidative stress	Reactive oxygen species (ROS) and Ca^2+^ concentration	Activation of ROS/MAPKs-mediated pathway.	MMQ and GH3 cells
Pawlikowski et al. (49)	Oxidative stress	Nitric oxide synthase (NOS)	NOS immunoreactivity is also detectable in all but two human pituitary adenomas and seems to negatively correlate with microvascularization.	Human tissue specimen and rats
Sabatino et al. (50)	Oxidative stress	Nuclear factor, erythroid 2 like 2 (Nrf2)	The evidence of oxidative stress in pituitary cells, accompanies by bigger and round mitochondria during tumor development, associates with augmented biogenesis and an increased fusion process.	Rats
Jaubert et al. (51)	Oxidative stress	Dopamine (DA)	(i) loss of mitochondrial potential; (ii) relocation of Bax to the mitochondria; (iii) cytochrome c release; (iv) caspase-3 activation, and (v) nuclear fragmentation, resulting in apoptosis.	GH3 cells
Onishi et al. (52)	Oxidative stress	The inducible NOS (iNOS)	Invasive adenomas have higher iNOS immunoreactivity, and this correlates with the MIB-1 labeling index.	Human tissue specimen
Huang et al. (53)	Oxidative stress	Nitric oxide (NO)	Nitric oxide mediates Nivalenol (NIV)-induced oxidative stress. Additionally, NIV induces caspase-dependent apoptosis, decrease in mitochondrial membrane potential and mitochondrial ultrastructural changes.	GH3 cells
Babula et al. (54)	Oxidative stress	Nitric oxide (NO) metabolites level in serum	The decrease of NO level after pituitary adenoma resection indicates the relationship between NO synthesis and pituitary adenoma occurrence.	Human
Guzzo et al. (55)	Apoptosis	Bcl-2 family	The intrinsic pathway (or mitochondrial) and extrinsic (or death-receptor pathway)	Rat pituitary cell lines, and human pituitaries tissue
Gottardo et al. (56)	Apoptosis	Humanin (HN) and Rattin (HNr)	Intratumor injection of BV-shHNr to nude mice bearing s.c. GH3 tumors increases the number of apoptotic cells, delays tumor growth, and enhances survival rate, suggesting that endogenous HNr may be involved in pituitary tumor progression.	GH3 cells
Gao et al. (57)	Apoptosis	Trefoil factor 3 (TFF3)	TFF3 protein level in pituitary adenoma is about 3.61 ± 0.48 folds of that in normal tissues (*P* < 0.01). After transfecting with small interference RNA (siRNA) against TFF3, the apoptotic ration is significantly elevated.	Human pituitary adenoma cell HP75
Tanase et al. (58)	Apoptosis	Apoptotic protease-activating factor-1 (APAF-1)	A bidirectional-inverted relationship between APAF-1 and cathepsin B expressions may result in changes in pituitary adenoma behavior.	Human tissue specimen
Yang et al. (59)	Apoptosis	MicroRNA-34a	miR-34a expression is significantly lower in GH4C1 cells, whereas miR-34a overexpression significantly inhibits GH4C1 cell proliferation and promotes cell apoptosis though SRY-box 7 (SOX7).	Rats
Cui et al. (60)	Apoptosis	MicroRNA-21	MiR-21 targets 3'-UTR of PITX2 gene to inhibit its expression. The elevated miR-21 and/or silencing PITX2 significantly depress PITX2 expression in HP75 cells, potentiate caspase-3 activity, decrease cell proliferation, and facilitate apoptosis.	Human tissue specimen
Wang et al. (39)	Apoptosis	MicroRNA-200c	MicroRNA-200c expression was inversely associated with Pten expression and facilitated apoptosis.	GH3 cells
Gong et al. (61)	Apoptosis	Adrenocorticotrophic hormone	UA inhibits the viability, and induces apoptosis of AtT20 cells, and decreases ACTH secretion.	AtT20 cells
Deyu et al. (36)	Autophagy	T-2 toxin	T-2 toxin induces abnormal cell morphology, cytoplasm and nuclear shrinkage, nuclear fragmentation and formation of apoptotic bodies, and autophagosomes.	GH3 cells
Kim et al. (37)	Autophagy	Cyclosporine A	Bcl-2 levels showed drug dose-dependent augmentation in autophagy and were decreased in apoptosis.	GH3 cells
Leng et al. (38)	Autophagy	Dopamine agonists	The increasing Reactive oxygen species (ROS) inhibited the MTOR pathway, induced macroautophagy/autophagy, and led to autophagic cell death (ACD) *in vitro* and *in vivo*.	Human pituitary tumor cell
Tagliati et al. (62)	Tumor immune	Presequence translocase associated motor 16 (MAGMAS)	Mitochondria-associated protein is involved in granulocyte-macrophage colony-stimulating factor signal transduction.	Human tissue specimen and AtT-20 D16v-F2 cells

## Mitochondrial Dysfunction-Mediated Reprogramming Energy Metabolism

Energy metabolism alterations are an emerging hallmark in tumor, which are still an unresolved issue that how energy metabolism system plays in formation and progression of tumors or metastases. Tumor cell energy metabolism has mainly focused on glucose metabolism and lipid metabolism. The metabolism of glucose to lactic acid in the presence of oxygen have been recognized in cancer cells, commonly called the Warburg effect (63). Further, the reverse Warburg effect put forward in 2009 provides complementary mechanisms for cancer energy metabolism (64). In addition, novel evidence is shedding light on alterations in lipid metabolism-associated pathways that have been discussed for past years. A gene expression study find that the upregulated lipogenesis pathways are associated with poor survival outcomes (65), and the elevated levels of lipid droplets are associated with cancer aggressiveness, which has been proposed to predict prognosis of cancer (66). More interesting is that lipids could be transferred from adipocytes to cancer cells by co-culture condition to promote cancer cell growth (67). It clearly indicates that lipid metabolism disorder is closely associated with tumorigenesis, whose study would be significantly enhanced with the development of lipidomics based on electrospray ionization/mass spectrometry (ESI-MS) (68).

**Figure 2:**
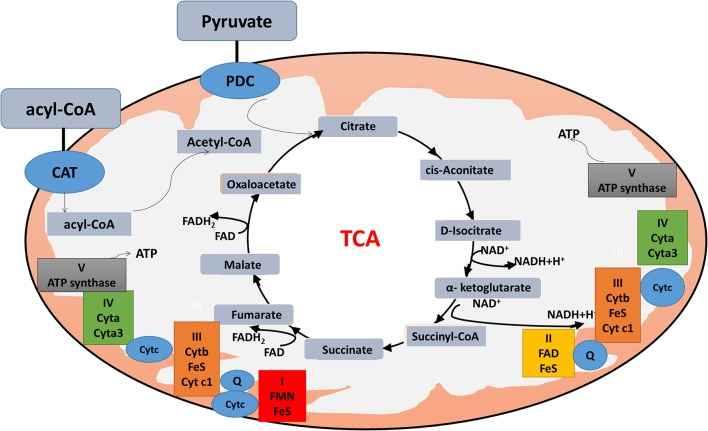
Mitochondrial physiology. Acetyl-CoA enters the mitochondrion via pyruvate or fatty acids. Pyruvate is imported through the mitochondrial inner membrane by the pyruvate dehydrogenase complex (PDC), and is oxidatively decarboxylated to produce acetyl-CoA. Fatty acids form acyl-CoA in the cytosol, and are transported into mitochondrion through carnitine (CAT) for β-oxidation. Acyl-CoA enters the citric acid cycle, and generates NADH and FADH2 (co-enzymes used in the electron transport chain) to produce ATP.

The citric acid cycle, oxidative phosphorylation (OXPHOS), and fatty acid beta-oxidation occur in the matrix of the mitochondrion in eukaryotic cells. Mitochondrial dysfunction is closely associated with energy metabolism reprogramming to associate cancer formation or progression (69). In the Warburg and reverse Warburg effects, cancer cells have metabolic symbiosis with mesenchymal cells, especially cancer-associated fibroblasts (CAFs), namely cancer cells produce reactive oxygen species (ROS) to induce oxidative stress and aerobic glycolysis of CAFs; in turn, CAFs produce lots of nourishments (especially lactate and pyruvate) to feed the adjacent cancer cells to produce more ATPs (64). The reverse Warburg effect shows metabolic interplay between high glycolytic cells and mitochondrial OXPHOS activates cells via lactate shuttle. Important enzymes involved in mitochondrial OXPHOS include the enzymes in the citric acid cycle and electron transport chain. The citric acid cycle is a series of enzymatic reactions to release energy through the oxidation of acetyl-CoA into ATP and carbon dioxide. The citric acid cycle is undergoing 10 steps to complete ATP production with a series of enzymatic reactions, including citrate synthase, aconitase, isocitrate dehydrogenase, α-ketoglutarate dehydrogenase, succinyl-CoA synthetase, succinate dehydrogenase, fumarase, and malate dehydrogenase. OXPHOS is the metabolic pathway in which cells use enzymes to oxidize nutrients for releasing energy to produce ATP. The eukaryotic electron transport chain contains NADH-coenzyme Q oxidoreductase (complex I), succinate-Q oxidoreductase (complex II), Q-cytochrome c oxidoreductase (complex III), cytochrome coxidase (complex IV), and ATP synthase (complex V). Furthermore, fatty acid molecules are broken down in the eukaryotic mitochondria to transform into acetyl-CoA to enter the citric acid cycle, and generate NADH and FADH2 that are co-enzymes used in the electron transport chain (Figure 2).

Mitochondria are the main location of energy metabolism pathways (citric acid cycle, OXPHOS, and fatty acid beta-oxidation). Some mitochondria-associated proteins have been reported to play a critical role in pituitary adenomas. For example, overexpression of lactate dehydrogenase A significantly promotes proliferation and invasion of pituitary adenomas, and positively correlates with higher Ki-67 index (41). Mutant succinate dehydrogenase in the citric acid cycle occurs in the pituitary adenomas (42). In addition, DNA sequencing-based genotypic studies demonstrate identical IDH1 mutations (c.394 C > T) in pituitary adenomas tissues (43). The high frequency of respiratory complex I mutations are found in mitochondrial DNA in a large panel of oncocytic pituitaries, which indicates dysfunction of respiratory complex I to cause instability of HIF1alpha in pituitary adenomas. Briefly, mutations in the mitochondria-coded MT-ND1 gene, an important composition of respiratory complex I, is closely associated with energy metabolic impairment to influence balance of succinate and alpha-ketoglutarate, which leads to the abnormal citric acid cycle metabolites (succinate and alpha-ketoglutarate) to be responsible for HIF1alpha stabilization in pituitary adenomas (44). The multifunctional succinate dehydrogenase (SDH) is located in the inner membrane of mitochondria, and serves as a critical step in Krebs cycle and a crucial member of the respiratory chain. SDH subunit D (SDHD) mutation is found in an aggressive GH-secreting pituitary adenoma, indicating SDHD mutation might link to the progression of pituitary adenomas (45). The study on SDH subunit B (SDHB) (+/–) mice finds that SDHx-deficiency is a main initiator to result in the cascade of molecular events for the formation of pituitary adenomas (46). The whole-exome sequencing analysis of pituitary oncocytomas found mitochondrial DNA mutations, respiratory complex I dysfunction, and reductions of lactate and lactate dehydrogenase A (LDHA) (48). Besides glycometabolism change, lipid metabolism is also alerted in pituitary adenomas. The gene microarray analysis of miRNAs expression profile between invasive and non-invasive non-functional pituitary adenomas finds that fatty acid metabolism plays a prominent role in pituitary adenomas (47). Therefore, energy metabolism alteration plays important roles in pituitary adenomas with high metabolic demand, which also influences cell proliferation, growth, and angiogenesis. The development of new drugs targeted mitochondria might be an new approach to block energy metabolism pathways for effective treatment of pituitary adenomas (70).

## Mitochondrial Dysfunction-Mediated Oxidative Stress

Oxidative stress reflects an imbalance between free radical/reactive oxygen/nitrogen species (ROS/RNS) productions and endogenous antioxidant defense mechanisms in the cells and body, which results in damage to proteins, DNA, membrane, and cellular organelles so on (71). ROS/RNS can be beneficial because they take part in attacking and killing pathogens by the immune system (72). Short-term oxidative stress might also be meaningful in prevention from aging (73). However, oxidative stress is also involved in the development of various diseases including cancers (74–79). Severely oxidative stresses even cause cell death, apoptosis, necrosis, cell migration, fibrosis, and angiogenesis. The lipid peroxidation of fatty acids as a type of oxidative stress increased ROS/RNS to injury bilayer lipid membranes. The secondary products of lipid peroxides such as malondialdehyde, aldehydes, 4-hydroxynonenal (HNE), hexanal, or acrolein have very long and broad effects (80).

**Figure 3:**
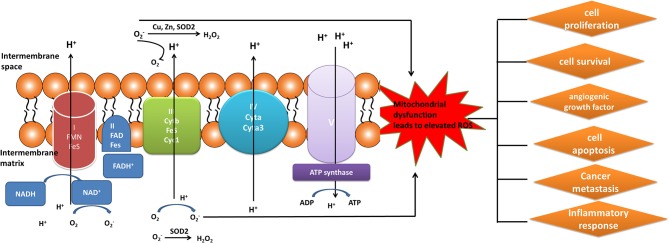
Mitochondrial generation of ROS. Complexes I, II, and III (complexes located on electron transport chain) play a pivotal role in the generation of ROS during the process of oxidative phosphorylation. The increased ROS regulated cancer progression, angiogenesis, metastasis, and survival.

Elevated level of ROS was a key constituent in cancer survival and resistance to treatment. The mitochondrial OXPHOS system is the major sites where produce endogenous ROS, including OH and superoxide radicals (${\text{O}}_{2}^{.-}$) (81). Mitochondrial complexes I, II, and III play a crucial role in the generation of mitochondrial ROS. Electrons tend to be leaky at complexes I and III to cause the incomplete reduction of oxygen to generate a free radical such as superoxide (80). Mitochondrial dysfunction could result in the increased ROS in cancer cells to mediate tumor-related signaling pathways and activate pro-oncogenic signaling, which regulate cancer progression, angiogenesis, metastasis, and survival (Figure 3). Although increased ROS does not benefit the cancer cell survival, however, antioxidant substance system is also activated in cancer cells to help cancer cell death escape (82). Moreover, the presence of mitochondrial nitric oxide synthase (mtNOS) provided the opportunity to review complementary aspects of mitochondrial physiology. The mtNOS could cause the generation of a partial nitric oxide (NO) in mitochondria (83), besides large amounts of NO produced by inducible NOS (iNOS) in pathophysiological status (84). NO can react quickly with the superoxide radicals to generate more toxic peroxynitrite anion (ONOO^−^) or hydroxyl radical (^.^OH). Mitochondria, as generators and targets of NO, determine the steady-state of NO though modulating the rates of consumption and production at the subcellular levels. Thus, mtNOS plays a crucial role in this process, which was activated by calcium and transcriptional/translational regulation (85). Furthermore, NO production in mitochondria was still decided by subcellular localization of mtNOS due to post-translational modification or protein-protein interactions. Therefore, mitochondria could produce NO by temporospatial distribution of mtNOS, and receive NO signal to regulate mitochondrial events (86).

It needs further study for imbalance of ROS and RNS production resulted from mitochondria dysfunction in pituitary adenomas. Many studies found the presence of ROS/RNS in human pituitaries, and the increased activities of ROS/RNS in pituitary adenoma compared to control tissues (41, 49). Also, oxidative stress in pituitary adenoma cells is accompanied by mitochondria swelling during tumor development, and associated with an increased fusion process and augmented biogenesis. An activation of the nuclear factor erythroid 2 like 2 (Nrf2) pathway and the reduction of oxidative damage signals were also observed during tumor development, which might provide survival advantages to pituitary adenoma cells (50). ROS pathway tends to be a medium in human pituitary cells. The pro-apoptotic effects are regulated partly by the dopamine transporter in GH3 pituitary cell lines, and involve oxidative stress as well as ROS formation. The use of only dopamine to treat hypophysis cells found that intracellular ROS was increased rapidly, and antioxidant N-acetyl-L-cysteine effectively inhibited dopamine-induced ROS generation and apoptosis (51). These data clearly demonstrated that ROS formation was closely related to signaling pathways in pituitary adenomas to affect tumor biological behavior. Studies found that 18beta-glycyrrhetinic acid had significantly antitumor effects on pituitary adenomas because this drug activated mitochondria-mediated ROS-mitogen-activated protein kinase (MAPK) pathways to induce cell apoptosis in pituitary adenomas, and that these activating effects were attenuated in pituitary adenomas by pretreatment with N-acetyl-L-cysteine, a ROS inhibitor (29). Moreover, many studies found the presence of NOS in human pituitaries, and NOSs were markedly higher expressed in invasive relative to non-invasive pituitary adenomas (52). NO mediated oxidative stress in pituitary adenoma cell lines to induce caspase-dependent apoptosis (53). Another study found that serum NO concentration was significantly decreased after the surgery of patients with pituitary adenomas (*n* = 21), thus monitoring serum NO level after pituitary adenoma surgery might benefit the prediction of its occurrence (54).

Thereby, oxidative stress has been considered as one of essential factors to contribute in the pathogenesis of pituitary adenomas. However, its molecular mechanisms remain unclear. The previous studies have been provided clues to the mechanism; for example, “mitochondrial theory of aging” increased production of ROS with altered expression of caveolae (87). It is meaningful to explore the roles of oxidative stress-mediated apoptosis, ER stress, DNA damage, metabolism, autophagy, migration, and anticancer drugs. The in-depth understanding of the relationship between oxidative stress and mitochondrial dysfunction might benefit improvement of chemotherapeutic approaches based on ROS/RNS-modulating drugs in the treatments of pituitary adenomas.

## Mitochondrial Dysfunction-Mediated Cell Apoptosis Dysregulation

**Figure 4:**
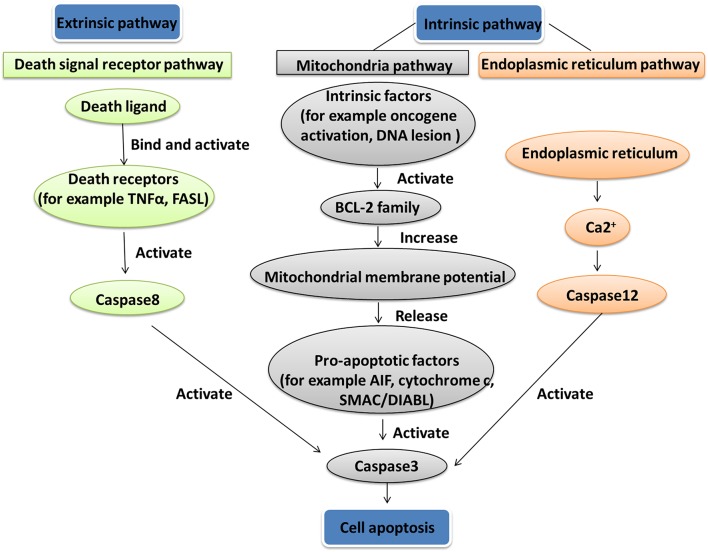
Apoptosis pathway. Two apoptosis activation mechanisms are the extrinsic and intrinsic pathways. The extrinsic pathway, including Fas path and TNF path, is activated by receptor-ligand-mediated model. The intrinsic pathway, including the mitochondrial apoptosis pathway and endoplasmic reticulum apoptosis pathway, is activated by intracellular signals.

Apoptosis is a gene-controlled form of programmed cell death, and is closely related to cancer. Apoptosis activation mechanisms include extrinsic and intrinsic pathways (88). The extrinsic pathway including FAS path and TNF path is activated by receptor-ligand-mediated model. Extracellular ligands binding to membrane death receptors result in the formation of death-inducing signaling complex (89). The intrinsic pathway including mitochondrial apoptosis and endoplasmic reticulum apoptosis pathways is activated by intracellular signals. Internal mitochondrial apoptosis pathway could be activated by internal apoptosis stimulators, such as persistent DNA damage, cell hypoxia, and cell growth factor deletion (90), or by death ligands and caspase 12. The Bcl-2 family proteins decrease the mitochondrial membrane potential promotion and increase mitochondrial outer membrane permeabilization, which leads to release pro-apoptotic factors such as AIF, cytochrome c, SMAC/DIABL, HTRA2/OMI, and ENDOG from the mitochondria into the cytosol (91). The increased mitochondrial outer membrane permeabilization is generally considered to activate the apoptotic pathway because the formation of apoptosome in the cytosol induces the caspase cascade (Figure 4) (92). Mitochondrial dysfunction occurred in a human pituitary tumor with mitochondrial morphological and functional changes, including large mitochondria, mitochondrial irregular swelling, and partly or fully disintegrated cristaes (16). Mitochondrial dysfunction caused change of mitochondrial membrane potential and internal apoptosis stimulator responses, which leads to mitochondria-mediated apoptosis signaling pathway alteration (93).

From the embryology angle, many apoptotic cells are formed in Rathke's pouch tissues from the roof of oral ectoderm at an early stage of pituitary gland formation. However, when adenohypophysis is formed in the distal part of the gland, the ratio of apoptotic cells was significantly lower than early stage. It means that an imbalance in apoptosis process might be the boundary between embryonic development and tumor progress. It is interested that pituitary adenoma cells undergo the imbalanced expressions of apoptosis-related genes/proteins to cause uncontrolled cell proliferation (55). Study found that targeting mitochondria could have an effective impact on the treatment of pituitary tumors through apoptosis pathway (56). For example, trefoil factor 3 (TFF3) is an apoptosis-related protein, and its knockout in human pituitary adenoma cell line decreased the levels of apoptosis-related proteins Bcl-2 and caspase-3, and increased the levels of Bax and cleaved caspase-3. It clearly demonstrated that TFF3 protein knockout can accelerate the apoptosis in human pituitary adenoma cells via mitochondrial apoptosis pathway (57). Moreover, apoptotic protease-activating factor-1 (APAF-1) is a pivotal functional protein to involve in the intrinsic mitochondrial apoptosis pathway. Low expression of APAF-1 was detected in most invasive pituitary adenomas, and was negatively correlated with the aggressive behavior of invasive pituitary adenoma, which suggested that shifting the balance of apoptosis mediators in cells could lead to changes of pituitary tumor behaviors (58). In addition, some microRNAs-target genes to mediate apoptosis pathway also have been found in pituitary adenomas. For example, tumor suppressor microR-34a overexpression significantly inhibited cell proliferation and promoted cell apoptosis in pituitary adenoma cells (59). miR-21 expression was lower in invasive relative to non-invasive pituitary adenoma tissues, and miR-21 targeted 3'-UTR of PITX2 gene to enhance caspase-3 activity, which inhibits cell proliferation and facilitates apoptosis in pituitary adenoma cells (60). Dysregulation of apoptosis-related proteins might be meaningful indicator of tumor progression because mitochondrial dysfunction pathway might facilitate tumorigenesis and tumor development (94).

The novel mechanisms in mitochondrial dysfunction-mediated cell apoptosis would facilitate the development of effective anti-cancer drugs. For example, the classical antitumor effect of paclitaxel is to target on tubulin in the cytoplasm. However, further study found that paclitaxel induced apoptosis by promoting the release of Cyt C after binding with Bcl-2 (95), which promoted one to accurately deliver paclitaxel though well-designed nanocarrier to improve its treatment performance (94). Furthermore, for adrenocorticotrophic hormone (ACTH)-producing pituitary adenomas (96), ursolic acid was found to be a potential agent targeting ACTH-producing AtT20 cells because ursolic acid inhibited cell proliferation, reduced ACTH secretion, and induced cell apoptosis in AtT20 cells with the decreased ratio of Bcl-2 to Bcl2-associated X protein to cause the release of mitochondrial cytochrome c from mitochondria to the cytosol, and activate subsequently caspase-9, -3/7, and -8. It indicates that ursolic acid may be a promising candidate drug for the treatment of ACTH-producing pituitary adenomas (61). Therefore, insights into mitochondria-mediated apoptosis might benefit the development of novel pro-apoptotic therapeutic drugs and discovery of biomarkers for early detection to treat a pituitary adenoma.

## Mitochondrial Dysfunction-Mediated Autophagy Dysregulation

Autophagy or autophagocytosis meaning “self-devouring” is the natural process and common cellular phenomenon, which is involved in the processes of phagocytosis and degradation of dysfunctional or unnecessary cell components, and also reusing of cellular components (97, 98). Briefly, the dysfunctional or unnecessary components are engulfed to form a double membrane called autophagosome, and then autophagosome is fused with the lysosome, followed by degradation of the contents into smaller constituents via acidic lysosomal hydrolase within lysosome (99). Autophagy takes part in various cellular functions, and particular attention has been paid to dual functions of autophagy in cancer—both protection cells against cancer and a potentially factor in cancer cell survival. Autophagy is regulated by many of the proteins, including oncogene and tumor suppressor proteins. Specifically, tumor suppressor proteins that negatively regulate mTOR pathway, such as LKB1, PTEN, TSC1/2, and AMPK, stimulate autophagy, while oncogenes that activate mTOR pathway, such as Ras, class I PI3K, AKT, and Rheb, inhibit autophagy, indicating the contribution of autophagy to cancer growth or tumor suppression. Moreover, the inhibition of autophagy induces genomic instability, oxidative stress, and tumorigenesis. Nevertheless, autophagy also functions as a protective factor under stress conditions, including nutrient starvation, and hypoxia that facilitates tumor cell survival and sensitivity and resistance to chemotherapy (100).

**Figure 5:**
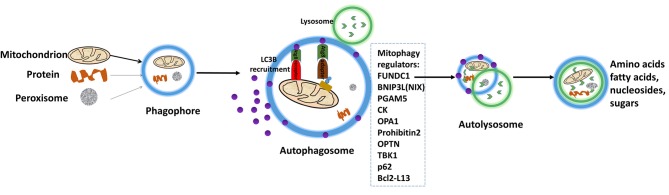
Depiction of the process of mitophagy. The engulfment of mitochondria forms a double-membrane-enclosed autophagosome, and then fuses with lysosomes. The process emits high-energy substances to recycle cell compartment, for example fatty acids and amino acids. Modified from Li et al. (15), with permission from Bioscientifica Limt.

Mitophagy is the complex biological process that cells selectively eliminate mitochondria by autophagy. The engulfment of mitochondria forms a double-membrane-enclosed autophagosome and then fuses with lysosomes. The process emits high-energy substances to recycle cell compartment, including fatty acids and amino acids (Figure 5) (101). Defective mitochondria undergoing damage or stress tend to the induction of mitophagy. However, the occurrence of mitophagy is not only restricted to the defective mitochondria but also involves normal ones (102). Mitophagy promotes turnover and the selective degradation of mitochondria, and prevents accumulation of dysfunctional mitochondria, which can lead to keep steady-state mitochondrial turnover, cellular metabolic needs, and certain cellular developmental stages (102). In this process, mitophagy depends on the general autophagy mechanism, meanwhile, both “mitophagy adaptors” and regulatory molecules are involved, such as p62, FUNDC1, prohibitin2, BNIP3L (NIX), PGAM5, OPA1, TBK1, CK, OPTN, and Bcl2-L13 (103). Those autophagy-related proteins activate downstream mitophagy pathways by post-translational modifications (104). Two distinct principal mitophagy mechanisms had been proved in mammalian cells. Firstly, receptor-mediated mitophagy is activated, and then recruits Atg8-like proteins to mitochondria to increase combination. Secondly, the highly ubiquitylated mitochondrial outer membrane proteins recruit bifunctional adapter proteins, which in turn increases the binding of Atg8-like proteins (105). Atg8-like proteins prompts encapsulation of mitochondria into autophagosomes through the expansion of the phagophore membrane; and fusion with the lysosome results in the formation of autolysosomes facilitating degradation of selected dysfunctional mitochondria (106). Also, mitophagy receptors exist some complex regulation mechanisms. An unmodified receptor is phosphorylated by a kinase, activating or deactivating downstream mitophagy pathway by increasing or decreasing Atg8-like proteins binding. This effect can be reversed by phosphatases. For NIX (BNIP3L), BCL2L13, and BNIP3, only the activated phosphorylation and the modified residue mechanism are understood but the kinases or phosphatases have not been identified yet for FUNDC1, the activated and deactivated phosphorylation intracellular mechanisms, the modified residues and participated kinases or phosphatases are well-known (107).

Mitochondrial dysfunction might affect mitophagy that can be related to metabolic reprogramming, inflammatory signaling, cell fate determination and differentiation, DNA damage responses in response to stress, which in turn lead to human disease incidence and etiology, including malignant tumor (103). It is well-known that mitophagy and mitochondrial dysfunction are related to pituitary adenomas. The mitochondrial toxicity and protective mechanisms of T-2 toxin are not fully understood in mammalian cells (108). The investigation of the cellular and mitochondrial toxicity of T-2 toxin shows that T-2 toxin significantly increases mitophagic activity, ROS and DNA damage in rat pituitary GH3 cells. With the increased expression of mitophagy-specific proteins, including E3 ubiquitin ligase Parkin, NIP-like protein X (NIX), and PTEN-induced putative kinase protein 1 (PINK1), T-2 toxin can be increased (109). The regulating mechanism of mitophagy is also mediated by nuclear factor (erythroid-derived 2)-like 2 (Nrf2)/PINK1/Parkin pathway in pituitary GH3 cells. The relevant drug activates the protective protein kinase A signaling pathway, which activates the Nrf2/PINK1/Parkin pathway to mediate mitophagy. Taken together, increasing mitophagy and mitochondrial dysfunction might increase chemo-resistance in pituitary GH3 cells (36). Sometimes apoptosis and autophagy coexist. Dopamine agonists such as bromocriptine and cabergoline have been successfully used in the treatment of pituitary prolactinomas. DRD5 activation increases production of ROS, inhibits the MTOR pathway, induces macroautophagy/autophagy, and leads to autophagic cell death (ACD) in human pituitary tumor cells (38). In addition, when cyclosporine A (CsA) induces apoptotic and ACD in pituitary GH3 cells, Bcl-2 levels show dose-dependent augmentation in autophagy and are decreased in apoptosis (37).

Autophagy can promote survival of tumor cells in starvation mode, or degrade cell apoptotic mediators to maintain the tumor clone. In such cases, treated patients with the late stage of autophagy—blockers (such as chloroquine), on the cells that depend on autophagy to survive, may be one of viable therapeutic measurements in fighting cancer (110). Thus, the qualities of mitophagy can be used as a therapeutic method for cancer prevention. Mitophagy plays a role in tumor suppression and tumor cell survival. One strategy is to induce mitophagy and enhance the function of antitumor. The other strategy is to inhibit mitophagy and thus induce apoptosis (111). The first strategy has been tested by monitoring dose-response anti-tumor effects during autophagy-targeted therapies. These treatment effects have shown that autophagy has some dose-dependence in tumor suppression and tumor cell survival progression. The result supports the development of therapies through autophagy (112). In addition, inhibition of the protein related to autophagy pathways may also serve as an anticancer therapy (113). Autophagy is a protein degradation system to play a role in maintaining homeostasis and inducing apoptosis. Thus, sometimes inhibition of autophagy has on the probability of existential risk as it may lead to tumor development instead of the desired cell death (114).

## Mitochondrial Dysfunction-Mediated Tumor Immunity

Tumor immune escape is an important hallmark in cancer (115). Tumor-evading immune destruction is closely correlated with prognosis or survival in various tumors (116). A study found a number of tumor immunity-related inflammatory cells, for example, cytotoxic T cells (CTLs), regulatory T cells (Tregs), myeloid-derived suppressor cells (MDSCs), and natural killer (NK) cells (37). It also found a number of tumor immunity-related pathways, such as altered interleukin signaling (117), MHC-I pathway (118), type 1 cytokine-induced T-cell (119), interferon gamma signaling (120), type I interferon-mediated responses (121), and transcription factor nuclear factor-kappa B (122). Along with the advancement in tumor immunology, the immune-checkpoint blockade therapy has been an important aspect in the mode of combined therapy of tumor. One of the most important immune checkpoint pathways has been applied between the PD-1 receptor expressed on activated T cells and its ligands, programmed death-1 ligand (PD-L1) and PD-L2 (123).

**Figure 6:**
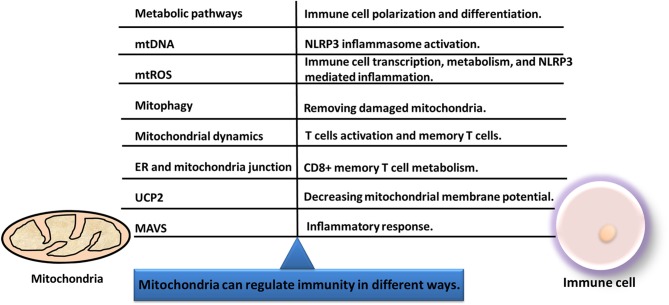
Immunity and mitochondria are interlinked with each other. Mitochondria can regulate immunity in different ways, including metabolic pathways, mitochondrial dynamics, mtDNA, mitophagy, mtROS, MAVS, UCP2, and ER-mitochondria junction.

Immunity process is interlinked with mitochondrial function. Mitochondria can regulate immunity in different ways (Figure 6): (i) Current literature shows that many changes of cancers occurred in substance metabolism pathways such as TCA cycle, oxidative phosphorylation, fatty acid oxidation, and amino acid metabolism. Mitochondria that induce transcriptional key enzymes or important molecule changes can lead to completely different results in immune cells (116). Thus, mitochondria can regulate differentiation, activation, and survival of immune cells (124). (ii) Mitochondrial DNA (mtDNA) could translocate from the mitochondria to cytoplasm and activate the NLRP3 inflammasome to induce IL-1β and IL-18 release (125). (iii) Mitochondria can transmit signals through mtDNA or mitochondrial ROS (mtROS) to regulate gene expressions of immune cells (126). (iv) Mitophagy is crucial for degradation of the damaged mitochondria, and the decreased mitophagy causes ROS increasing which further makes the susceptibility to infections (127). (v) Immune functions are influenced by fission and fusion of mitochondria, which determines mitochondrial mobility and mass. (vi) When mitochondria are located near endoplasmic reticulum (ER), the mitochondria and ER junction signaling could be activated in immune cells to influence immune cell metabolism (128). (vii) The inflammatory response can be initiated by mitochondrial antiviral signaling (MAVS). Therefore, mitochondrial machinery is crucial for immune functions, such as metabolic pathways, mtDNA, mtROS, mitochondrial dynamics, and mitophagy.

Tumor immune microenvironment is gradually recognized as a critical contributor in tumor progression, development, and control. The increasing studies show that immune cells infiltrate in pituitary adenomas. Dysregulation of several genes in granulocyte-macrophage colony-stimulating factor signal transduction has been regarded as a possible alteration underlying the occurrence and development of pituitary tumors, such as mitochondria-associated protein Tim 16. High-expression of Tim 16 is identified in mouse and human ACTH-secreting pituitary adenomas compared to normal pituitary to protect pituitary cells from apoptosis (62). Also, more macrophages are identified in larger pituitary adenomas, and more T cells are detected in GH-secreting pituitary adenomas. A positive correlation is found between the numbers of CD68+ macrophages and tumor sizes and grades for pituitary adenoma invasiveness. The density of infiltrated CD4+ and CD8+ T-lymphocytes may be relatively insufficient in these pituitary adenomas, but CD4+ and CD8+ T lymphocytes are significantly more in GH-secreting adenomas than non-GH adenomas. Both densely and sparsely granulated GH-adenomas had significantly more CD4+ cells than ACTH-adenomas, and significantly more CD8+ cells than null cell adenomas. These results suggest an association of the enhanced T-lymphocytes infiltration and invasiveness in pituitary adenomas, and that adjuvant immunotherapy might block the tumor enlargement and invasiveness of pituitary adenomas (129). Furthermore, study shows that low expression levels of immune-related genes induce the occurrence of pituitary adenomas (130). Another study show reveals the association of NF-κB, MMP-9, and MICA in pituitary adenomas, the higher expressions of MICA, MMP-9, and NF-κB in mRNA and protein levels in pituitary adenomas relative to healthy tissues; which found that the upregulation of NF-κB can activate the expression of MICA and increase MMP-9 expression to hydrolyze MICA into sMICA to facilitate tumor immune escape (131). Although most pituitary adenomas are treated successfully, it remains challenging to treat invasive non-functional pituitary adenomas as well as functional pituitary adenomas unresponsive to traditional therapy. Immunotherapy might be a potential alternative therapy for pituitary tumors that are resistant to traditional therapy (132). The positive PD-L1 immunostaining is significantly more frequent in functional relative to non-functional pituitary adenomas (*p* = 0.000). The expression level of PD-L1 is more related to the increased blood levels of ACTH-, PRL-, GH-, and cortisol-secreting pituitary adenomas. PD-L1 expression is also associated with GH and PRL immunostaining density and higher Ki-67 index (133). Thereby, immunotherapy might be a promising therapy option of functional pituitary adenomas on the basis of in-depth understanding of mitochondria-mediated immunity in pituitary adenomas.

## The Mitochondrial Dynamics in Cancer

**Figure 7:**
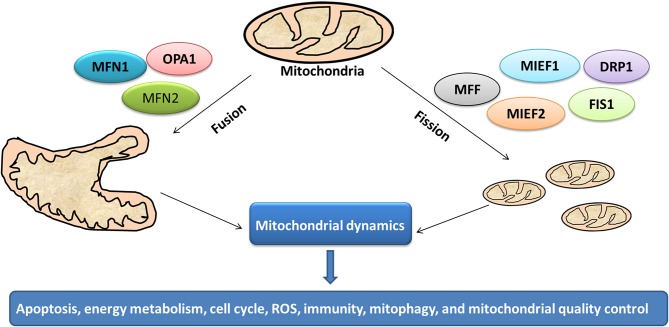
Mitochondrial dynamics in cancer. Cycles of fission and fusion are crucial for various cellular processes such as apoptosis, energy metabolism, cell cycle, ROS, immunity, mitophagy, and mitochondrial quality control.

Mitochondrion is a highly dynamic organelle under the coordination between fission and fusion cycles, which is referred as “mitochondrial dynamics.” Fission-fusion cycles affect mitochondria shape, size, and distribution (134). Mitochondria transient and rapid morphological adaptations are crucial for various cellular processes such as apoptosis (135), energy metabolism (136), cell cycle (137), ROS (138), immunity (139), mitophagy (140), and mitochondrial quality control (Figure 7). Mutations of the key machinery components or defects of mitochondrial dynamics are associated with lots of human diseases, including cancer (141). These dynamic transitions are primarily regulated by large GTPases. Mitochondrial fission and fusion cycle is a multi-step process. Mitochondrial fission is controlled by recruitment of dynamin-related protein 1 (Drp1) by adaptors at ER- and actin-mediated mitochondrial constriction sites. Drp1 oligomerization results in mitochondrial constriction, which leads to dynamin 2 recruitment to terminate mitochondrial outer membrane scission. Inner mitochondrial membrane constriction is an independent process, and mediated by calcium influx. Mitochondrial fusion is driven by mitofusins 1 and 2 within the outer mitochondrial membrane, and mediated by optic atrophy 1 with inner membrane (142). Moreover, several members of membrane lipid composition could undergo post-translational modifications to regulate these processes (143). Therefore, it is crucial for one to in-depth understand molecular mechanisms of mitochondrial dynamics for further studying various cellular processes associated with human diseases.

Various cellular processes, such as apoptosis (135), energy metabolism (136), cell cycle (137), ROS (138), immunity (139), and mitophagy (140), are closely related to mitochondrial dynamics and mitochondrial dysfunction. The relationship study between cell apoptosis and mitochondrial fission found that IR-783 induces Drp1 translocation from cytoplasm to mitochondria, makes the expression of mitochondrial fission proteins (MFF) and mitochondrial fission factor fission-1 (Fis1) increased, and the expression of optic atrophy 1 (OPA1) and mitochondrial fusion proteins mitofusin1 (Mfn1) decreased. The process of mitochondrial translocation of Drp1 mediated mitochondrial fission and markedly induced apoptosis *in vivo* and xenograft model (144). The signaling pathways involved in mitochondrial dynamics regulation and their roles in maintaining energy metabolism has become an active area of research. Mitochondrial dynamic events, such as fusion, fission, and transport, affect the mitochondrial shape, size, function, and subcellular localization. Mitochondria dynamic changes play a crucial role in assisting metabolite transfer, biogenesis, and degradation to maintain energy homeostasis (136). Mitochondrial electron transport chain-derived ROS and mitochondrial fission/fusion rates influence this delicate balance between mitochondrial dynamics and mitochondria-derived ROS production, which plays main roles in malignant diseases (138). In addition, mitochondrial dynamics role in cancer growth connects with the immune system activity, especially T cells. Although it has not been directly verified whether or not mitochondrial dynamics are associated with lymphocytes memory formation, Drp1-dependent mitochondrial fission has the potential contribution to regulate NK memory phase. It is indicated that mitochondrial dynamics is also possible to play role in the cytotoxic activity of these lymphocytes against cancer. Thereby, mitochondria can control local calcium influx to regulate the inner mitochondrial membrane constriction. It would be then interesting to see how mitochondrial dynamics to regulate the release of cytotoxic granules by T lymphocytes (139). The close interactions between mitochondrial dynamics and mitophagy become main players in the physiological cell processes in cancers. The new metabolic changes that mainly lead to mitochondrial functions and dysfunctions are strongly related to cancers, mitochondrial dynamics, and mitophagy (140). Study demonstrates that the BCL2/BCLXL inhibitor ABT737 mediates intrinsic apoptotic pathways and mitophagy through increasing levels of DRP1 in mitochondria and rates of mitochondrial fission (145).

## Conclusion

In spite of considerable progresses in understanding mitochondrial dysfunction pathway networks and mitochondrial dynamics in the pathogenesis of pituitary adenomas, many key issues remain unclear. Several lines of evidence indicate that mitochondrial dysfunction emerge cross-links with various complex biological processes, including energy metabolism, oxidative stress, cell apoptosis, cell cycle, mitophagy, and immunity process. Moreover, mitochondrial dynamics is closely associated with mitochondrial dysfunction, and also plays a critical role in many biological processes. This review breaks new ground in the comprehensive understanding of potential mechanisms underlying between mitochondrial homeostasis and tumorigenesis in pituitary adenomas. The association of mitochondrial dysfunction, mitochondrial dynamics, and the complex biological processes helps to broaden the knowledge of mitochondrial functions in cancer and perspectives regarding tumor treatment. It offers the new promising to develop new candidate targets based on mitochondrial dysfunction pathway and mitochondrial dynamics, for effective therapy in pituitary adenomas.

## Author Contributions

NL collected and analyzed references, prepared figures and tables, and wrote the manuscript. XZ conceived the concept, designed, coordinated, critically revised/wrote manuscript, and was responsible for its financial supports and the corresponding works. All authors approved the final manuscript.

### Conflict of Interest

The authors declare that the research was conducted in the absence of any commercial or financial relationships that could be construed as a potential conflict of interest.

## Abbreviations

ACTH: Adrenocorticotrophic hormone
AIF: Apoptosis-inducing factor mitochondria-associated 1
AMPK: Protein kinase AMP-activated catalytic subunit alpha 1
APAF-1: Apoptotic protease-activating factor-1
ATP: Adenosine triphosphate
Bcl-2: Apoptosis regulator
BNIP3L: BCL2 interacting protein 3 like
CAF: Cancer-associated fibroblasts
CAPN2: Calpain 2
CK: Choline kinase alpha
CTLs: Certain cytotoxic T cells
DNA: Deoxyribonucleic acid
Drp1: Dynamin-related protein 1
ER: Endoplasmic reticulum
ESI-MS: Electro-spray ionization-mass spectrometry
FADH2: Flavin adenine dinucleotide reduced
Fis1: Mitochondrial fission factor fission-1
FUNDC1: FUN14 domain containing 1
GH: Growth hormone
HNE: 4-hydroxynonenal
HTRA2/OMI: HtrA serine peptidase 2
IDH1: Isocitrate dehydrogenase 1
IL-18: Interleukin-18
IL-1β: Interleukin 1β
ITPR1, Inositol 1, 4: 5-trisphosphate receptor type 1
KIi-67: Nuclcar-associated antigen
LDHA: Lactate dehydrogenase A
LKB1: Serine/threonine kinase 11
MAVS: Mitochondrial antiviral signaling
MDSCs: Myeloid-derived suppressor cells
MFF: Mitochondrial fission proteins
Mfn1: Mitochondrial fusion proteins mitofusin 1
MHC-I, Major histocompatibility complex: class I
MMP-9: Matrix metallopeptidase 9
MT/ND1: Mitochondrially encoded NADH dehydrogenase 1
mtNOS: Mitochondrial nitric oxide synthase
mtROS: Mitochondrial ROS
NADH: Nicotinamide adenine dinucleotide
NF-κB/IRF: Nuclear factor kappa B subunit 1/tripartite motif containing 63
NK: Natural killer
NLRP3: NLR family pyrin domain containing 3
NO: Nitric oxide
Nrf2: Nuclear factor erythroid 2 like 2
OPA1: Optic atrophy 1
OPTN: Optineurin
OXPHOS: Oxidative phosphorylation
PD-L1: Programmed cell death 1 ligand 1
PD-L2: Programmed cell death 1 ligand 2
PGAM5: PGAM family member 5
PI3K, Phosphatidylinositol-4: 5-bisphosphate 3-kinase catalytic subunit beta
PINK1: PTEN-induced putative kinase protein 1
PITX2: Paired like homeodomain 2
PPP3CA: Protein phosphatase 3 catalytic subunit alpha
PPPM, Predictive, preventive: and personalized Medicine
PRKCA: Protein kinase C alpha
PTEN: Phosphatase and tensin homolog
Ras: RAS proto-oncogene GTPase
Rheb: Ras homolog mTORC1 binding
RIG-I: Retinoic acid-inducible gene I
RNA: Ribonucleic acid
RNS: Reactive nitrogen species
ROS: Reactive oxygen species
SDH: Succinate dehydrogenase
SIRT3, Programmed cell death 1PD-1: sirtuin 3
SMAC/DIABL: Diablo IAP-binding mitochondrial protein
TBK1: TANK binding kinase 1
TCA: Tricarboxylic acid cycle
TFF3: ENDOG trefoil factor 3
TLR2: Toll-like receptor 2
TNF: Tumor necrosis factor
Tregs: Regulatory T cells
TSC1/2: TSC complex subunit $\frac{1}{2}$.
